# Exploitation of heterosis loci for yield and yield components in rice using chromosome segment substitution lines

**DOI:** 10.1038/srep36802

**Published:** 2016-11-11

**Authors:** Yajun Tao, Jinyan Zhu, Jianjun Xu, Liujun Wang, Houwen Gu, Ronghua Zhou, Zefeng Yang, Yong Zhou, Guohua Liang

**Affiliations:** 1 Jiangsu Key Laboratory of Crop Genetics and Physiology/Co-Innovation Center for Modern Production Technology of Grain Crops, Key Laboratory of Plant Functional Genomics of the Ministry of Education, Yangzhou University, Yangzhou 225009, China; 2 Institute of Food Crops, Jiangsu Academy of Agricultural Sciences, Nanjing 210014, China

## Abstract

We constructed 128 chromosome segment substitution lines (CSSLs), derived from a cross between *indica* rice (*Oryza sativa* L.) 9311 and *japonica* rice Nipponbare, to investigate the genetic mechanism of heterosis. Three photo-thermo-sensitive-genic male sterile lines (Guangzhan63-4s, 036s, and Lian99s) were selected to cross with each CSSL to produce testcross populations (TCs). Field experiments were carried out in 2009, 2011, and 2015 to evaluate yield and yield-related traits in the CSSLs and TCs. Four traits (plant height, spikelet per panicle, thousand-grain weight, and grain yield per plant) were significantly related between CSSLs and TCs. In the TCs, plant height, panicle length, seed setting rate, thousand-grain weight, and grain yield per plant showed partial dominance, indicating that dominance largely contributes to heterosis of these five traits. While overdominance may be more important for heterosis of panicles per plant and spikelet per panicle. Based on the bin-maps of CSSLs and TCs, we detected 62 quantitative trait loci (QTLs) and 97 heterotic loci (HLs) using multiple linear regression analyses. Some of these loci were clustered together. The identification of QTLs and HLs for yield and yield-related traits provide useful information for hybrid rice breeding, and help to uncover the genetic basis of rice heterosis.

Food shortages are becoming a serious global problem because of the increasing population and decreasing area of available arable land. The world’s population is expected to reach 9 billion by 2050^1^, and the area of farmland per capita will be just 0.151 ha. Rice is one of the most important staple foods, feeding half of the world’s population. Improvements to rice yield will significantly contribute to addressing food security issues. Since the 1970s, the utilization of heterosis in inter-subspecies has provided an avenue for rice breeding^2^,^3^. The yield production reached 10.5 t/ha in ‘super-hybrid rice breeding’ Phase I in 2000, and increased to 12 t/ha and 13.5 t/ha in 2004 and 2012, respectively^4^. In China, hybrid varieties account for approximately 50% of all cultivated rice^5^.

Heterosis, also known as hybrid vigor, is defined as the superior performance of a hybrid relative to its parents, including yield and adaptability to abiotic/biotic environmental conditions^6^. Although this phenomenon is widely acknowledged in animal and plant breeding^7^,^8^,^9^, the genetic basis of heterosis remains unclear. Three classical hypotheses, including dominance^10^,^11^,^12^,^13^, overdominance^14^,^15^,^16^ and epistasis^17^,^18^ have been proposed to explain heterosis. Molecular markers and genetic linkage maps provide a means to identify loci for heterosis. Quantitative trait loci (QTLs) mapping has proved to be an informative approach for dissecting complex traits and heterosis in rice and other crops, including maize, cotton, and oilseed rape, and many QTLs for yield and other important agronomic traits have been detected. In earlier studies, some non-permanent segregating populations such as F_2_, F_2:3_, BC_1_F_2_ were used to reveal the genetic basis of heterosis. However, the phenotypic data from these populations are not be allowed to be repeated under multiple environmental conditions. To address this issue, some permanent populations, such as immortalized F_2_ and recombinant inbred lines (RILs), were developed to investigate heterosis loci.

A more accurate estimate of the effects of QTLs, including those for heterosis, can be obtained using advanced populations such as near-isogenic lines (NILs) and chromosome segment substitution lines (CSSLs). For instance, 21 heterosis loci with significant effects on hybrids were identified in rice using 66 CSSLs and their 66 corresponding F_1_ plants with the recurrent parent^19^. Fifteen QTLs contributing to heterosis of plant height were identified by comparing performance among 15 CSSLs, their hybrids, and the recurrent parent^20^. Recently, combining QTL mapping and transcriptome profiling, multiple QTLs for yield-component heterosis were characterized^21^. Among these QTLs, one major QTL *RH8* (*rice heterosis 8*) was cloned. Interestingly, *Ghd7*, which also controlled rice flowering-time, has been found contributed to heterosis in hybrid as well^22^. These results help to clarify the genetic basis of rice heterosis.

In the application of rice heterosis, the F_1_ offspring originate from hybridization between restorer lines/male parents and male sterile lines/female parents, rather than the parents themselves. Therefore, we generated three TC (testcross) populations by mating three photo-thermo-sensitive-genic male sterile (PTGMS) lines with a set of whole-genome-resequencing-genotyped CSSLs. The phenotypes of these populations were estimated and used to investigate heterosis loci for seven yield and yield-related traits through QTLs analyses.

## Results

### Performance of the populations

To identify QTLs for rice heterosis, three PTGMS lines (Guangzhan63–4s, 036s, and Lian99s) were used as females and were crossed with the 128 CSSLs to generate four testcross populations (TCs): TC 1 (Guangzhan63-4s × CSSLs in 2008), TC 2 (Guangzhan63-4s × CSSLs in 2010), TC 3 (036s × CSSLs in 2014), and TC 4 (Lian99s × CSSLs in 2014). We also generated F_1_ hybrids of Guangzhan63-4s × 9311, 036s × 9311, and Lian99s × 9311, which were tested along with the two parents.

**Figure 1:**
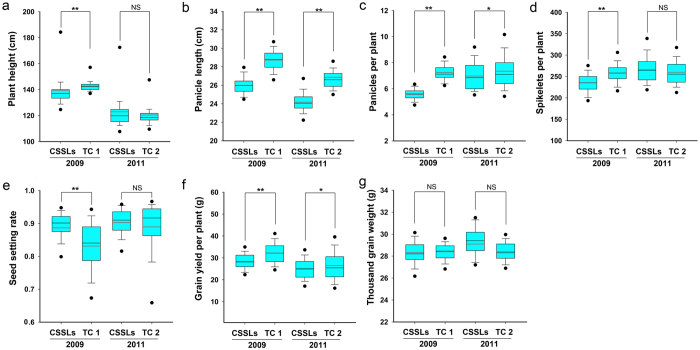
Means and ranges of yield-related traits measured in CSSLs, TC 1 in 2009, and TC 2 in 2011. (*p ≤ 0.05, **p ≤ 0.01, NS not significant).

**Figure 2:**
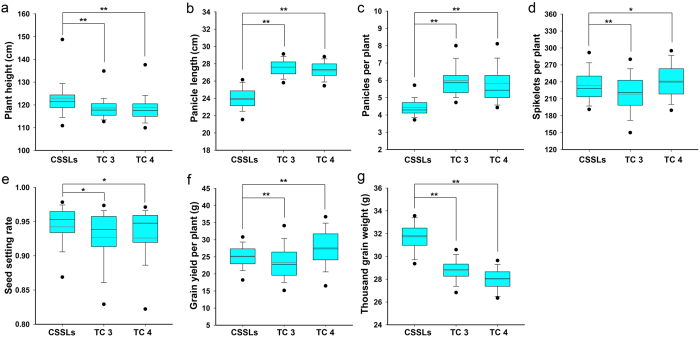
Means and ranges of yield-related traits measured in CSSLs, and TC 3 and TC 4 in 2015. (*p ≤ 0.05, **p ≤ 0.01, NS not significant).

Seven yield-related agronomic traits of the CSSLs and four TC F_1_ hybrids were measured in 2009, 2011, and 2015. The seven quantitative traits investigated were plant height (PH), panicle length (PL), panicle per plant (PPP), spikelet per panicle (SPP), seed setting rate (SSR), grain yield per plant (GYPP), and thousand-grain weight (TGW). The means and ranges of these seven quantitative traits are summarized in Figs 1 and 2. Genetic variations were observed for all traits in all three data sets.

In 2009, except for PH and SSR, the performance of all traits showed approximately normal distributions in the CSSLs. In the TC 1 population, PL, SPP, SSR, GYPP, and TGW data showed normal distributions (Table S1). We compared the phenotypic values between CSSLs and TC 1 hybrids. The means of most traits, except for SSR and TGW, in the TC 1 population were higher than the corresponding values in the CSSLs, suggesting that the heterozygous F_1_ was superior to the corresponding parental lines (Fig. 1, Table S4). In 2011, PL, SPP, PPP, and GYPP data showed normal distributions in the CSSLs. The PPP, SPP, GYPP, and TGW data showed normal distributions in the TC 2 population (Table S2). In addition, the mean values of PL, PPP, and GYPP in TC 2 were higher than those in the CSSLs in 2011 (Fig. 1, Table S5). Based on the combination of data from 2009 and 2011, we concluded that Guangzhan 63-4s might have a high general combining ability.

The data collected in 2015 were subjected to Kolmogorov–Smirnov (K–S) tests, which showed that only SPP, GYPP, and TGW had normal distributions in the CSSLs. However, except for PH, PPP, and SSR, the other traits showed normal distributions in the TC 3 and TC 4 populations (Table S3). Only PL and PPP showed better performance in the TC 3 and TC 4 populations than in the CSSLs. The mean values of SPP and GYPP in the CSSLs were higher than those in the TC 3 population, and lower than those in the TC 4 population (Fig. 2, Table S6).

These results suggested that different PTGMS may have different combination abilities and that Guangzhan63-4s and Lian 99s have higher general combination abilities than 036s for yield production.

### Relationships between traits in CSSLs and TCs

We conducted correlation analyses to determine the relationships for seven yield-related traits between the CSSLs and TCs and to discover whether each trait was correlated between TCs and their parental CSSLs. The correlation coefficients for the seven phenotypic values in CSSLs and TCs are summarized in Tables 1, 2, 3.

In the CSSL populations, significant correlations were detected between the mean values of PH and PL, and between the mean values of PH and GYPP, over the 3 years (Tables 1, 2, 3). Some traits were significantly correlated over 2 years, such as PH and SPP (2009, 2015) (Tables 1 and 3), GYPP and PPP (2011, 2015) (Tables 2 and 3), GYPP and SPP (2009, 2015) (Tables 1 and 3), and GYPP and SSR (2009, 2015) (Tables 1 and 3). Table 4 summarizes the correlation coefficients between the phenotypic values of the individual TCs and those of their parental CSSLs for the seven traits. Except for PPP and SSR, the other five traits were significantly correlated between the TCs and CSSLs. Among them, the correlation between PH and TGW was significant in all four TCs–CSSLs, while the correlation between SPP and GYPP was significant in three TCs–CSSLs.

### Detection of QTLs and heterotic loci

The estimated broad sense heritability of the seven traits ranged from 21.47% to 79.85% in the TCs (Table 5). Among the seven traits, PH, PL, GYPP, and TGW showed high heritability (>50%), indicating that genetic factors played dominant roles in these four traits. The lowest broad sense heritability was for PPP, suggesting that it was strongly affected by environmental factors. We also estimated narrow sense heritability to detect the additive effects for each trait. Additive effects mainly controlled PH, GYPP, and TGW. In general, additive effects are steadily inherited. Therefore, these three traits could be effectively selected for in early generations in hybrid rice breeding.

Among the 3 years, a total of 62 QTLs associated with yield-related traits were detected in the CSSLs, and 97 heterotic loci (HLs) were identified in four TC populations. Most of these QTLs and HLs explained more than 10% of total variation (Tables S7–13).

#### Plant height

For PH, 20 QTLs and 21 HLs were detected in the CSSLs and TCs, respectively (Table S7). Among the detected QTLs, *qPH1-1*, which was located in bin x36, was identified in all 3 years. This region contains the semi-dwarf gene *sd1*^23^. The QTL *qPH2-1*, located in bin x107 on chromosome 2, was detected in 2009 and 2015. The QTL *qPH2-2*, located in bin x108 on chromosome 2, was detected in 2009 and 2015. The HL *hPH1-4*, also located in bin x36, was identified in all TCs, and the HL *hPH8-1* in bin x278 was stably identified in TC 1 and TC 2.

#### Panicle length

Only five QTLs for PL were detected over the 3 years. Among them, three were on chromosome 1 and the other two were on chromosome 8 (Table S8). The QTL *qPL1-1*, located in bin x36, was identified in 2009 and 2015. The interval sizes of the two QTLs on chromosome 8, *qPL8-1* and *qPL8-2,* were just 154,545 bp and 103,885 bp, respectively, illustrating the advantage of CSSLs to narrow down regions of interest for further mapping of yield-related genes. In the TC populations, 16 HLs were identified on chromosomes 1, 2, 3, 5, 6, 8, and 11 (Table S8). One HL, *hPL1-2*, located in bin x36, was identified in all three TCs.

#### Panicles per plant

Four QTLs and 13 HLs were identified in the CSSLs and TCs, respectively (Table S9). Except for the HLs located in bin x275 in TC 1 and TC 3, there were no repeated bins found in all populations. These results indicated that PPP is a complicated trait that is significantly affected by environmental factors. The QTL with the largest effect was mapped to bin x279 and explained 39.89% of the phenotypic variance in PPP.

#### Spikelets per plant

Seven QTLs were detected for SPP. The QTL *qSPP1-1*, located in bin x36, was identified in all 3 years. Among the HLs, *hSPP1-1*, also located in bin x36, was identified in three TCs, and *hSPP6-1* and *hSPP6-2* were simultaneously detected in TC 3 and TC 4 (Table S10).

#### Seed setting rate

We identified 17 QTLs for SSR. Only *qSSR3-4*, located in bin x154; *qSSR3-5*, located in bin x155; and *qSSR6-1*, located in bin x232, were identified in 2 years. We identified 13 HLs for SSR on seven chromosomes and there were no repeatedly detected bins (Table S11). These results indicated that SSR is a complicated quantitative trait that is affected by environmental factors.

#### Thousand-grain weight

For the CSSLs, *qTGW3-1*, *qTGW6-1*, *qTGW6-2*, *qTGW6-3,* and *qTGW10-1* were detected over the 3 years. Among these QTLs, *qTGW6-1* was detected in 2011 and 2015. In the TCs, 10 HLs were identified on chromosomes 2, 4, 5, 6, and 8 (Table S12).

#### Grain yield per plant

The GYPP is an important agronomic trait, and QTLs and HLs associated with GYPP are highly significant for rice breeding and improving rice outputs. Four QTLs for GYPP (*qGYPP1-1*, *qGYPP6-1*, *qGYPP6-2,* and *qGYPP8-1*) were identified in all 3 years (Table S13); their interval sizes were 791,655 bp, 872,289 bp, 259,355 bp, and 538,176 bp, respectively. Twelve HLs underlying GYPP were identified in the four TCs. In TC 1, *hGYPP8-1* was identified in bin x278, the same bin harboring *qGYPP8-1* (Table S13).

Some bins harbored QTLs and HLs for several traits. For example, bin x36 harbored QTLs and HLs for PH, PL, SPP, and GYPP, suggesting pleiotropic characteristics or tight linkage among genes controlling these traits.

## Discussion

The grain yields of hybrid rice lines are 20~30% higher than those of conventional varieties. However, the yields have plateaued and progress in high-yielding hybrid rice breeding is slowing down. One of the key reasons is the limited genetic variability. *Indica* and *japonica* are the two main subspecies of cultivated rice. Significant molecular and physiological differences have been detected between these two rice groups^24^,^25^,^26^. One study confirmed that inter-subspecific hybrid rice lines have ~25% greater yield production than that of inter-varietal hybrid rice^27^. Consequently, it is important to identify QTLs for yield and yield-related traits in *indica* and *japonica* rice, and then pyramid them to advance hybrid rice breeding.

The production of CSSLs is a powerful strategy for precise QTLs mapping in a genomic region and to validate QTLs in earlier or segregating generations. CSSLs give a more accurate estimate of genetic effects in a specific background. Previously, we developed a set of CSSLs derived from the crossing and back-crossing of two sequenced rice cultivars: 9311, an elite *indica* cultivar, as the recipient, and Nipponbare, a *japonica* cultivar, as the donor. The 9311 variety is an elite line with excellent yield, quality, and resistance, and it has been widely used as the male parent of two-line hybrid rice in China. As a result, any improved lines in the CSSLs population due to the introgressed Nipponbare chromosome segments can be used directly to generate hybrid rice.

**Figure 3:**
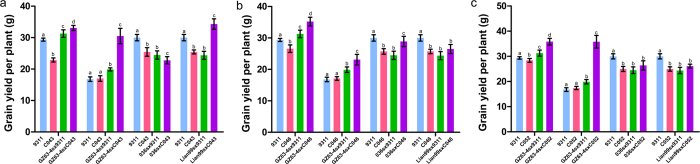
Phenotypic variation of GYPP in 9311, CSSLs, and their F_1_ hybrids. (**a**) Phenotypic variation of GYPP in 9311, C043, and their F_1_ hybrids; (**b**) Phenotypic variation of GYPP in 9311, C046, and their F_1_ hybrids; phenotypic variation of GYPP in 9311, C052, and their F_1_ hybrids. Different letters following mean values indicate significant difference (*P* ≤ 0.05, Tukey’s honestly significant difference test. GZ63-4s, Guangzhan 63-4s.

The genetic basis of rice heterosis is complicated, and many attempts have been made to identify key and valuable loci for hybrid breeding. In this study, to accurately identify useful loci for rice heterosis, three PTGMS lines were crossed with 128 CSSLs and 9311 to develop four sets of testcross populations. We totally found 62 QTLs and 97 HLs, and additional studies will be needed to fine-map these genes. Analyses of phenotypic data for seven traits across 3 years indicated that four traits (PH, SPP, GYPP, and TGW) were significantly related between CSSLs and TCs. In all four crosses, PH showed the highest correlation, suggesting that plant height of hybrid rice depends largely on male parent. However, it was difficult to predict the performance of PPP and SSR. Rice heterosis is usually detected in terms of yield production. As summarized in Table 4, the performance of the F_1_ hybrid was closely linked to that of the parent lines. That is, better parents, better hybrids. However, the grain yields of several combinations (Guangzhan63-4s × C043, Guangzhan63-4s × C046, and Guangzhan63-4s × C052) were significantly higher than that of Guangzhan63-4s × 9311 (Fig. 3). In contrast, the grain yield per plant in C043, C046, and C052 was lower than in 9311. This result highlights the complexity of rice heterosis, and indicates that overdominance may play an important role in specific rice hybrids.

Variance analysis showed that the average broad sense heritability of PH, TGW, and GYPP in four TCs was 79.85%, 76.75%, and 75.50%, respectively. Five traits (PH, PL, SSR, GYPP, and TGW) showed partial dominance, indicating that partial dominance is an important contributor to heterosis for yield-traits in rice (Table 5). Very recently, Huang *et al*.^28^ used integrated method to uncover the mechanisms of heterosis in different hybrid systems and found that the heterozygous state of these grain-yield QTLs always show positive partial dominance, which supports our results. Zhou *et al*.^29^ reported that overdominance/pseudo-overdominance is the most important contributor to heterosis of yield and number of grains per panicle. In our research, we also found that SPP showed overdominance, which is consistent with Zhou’s results.

**Figure 4:**
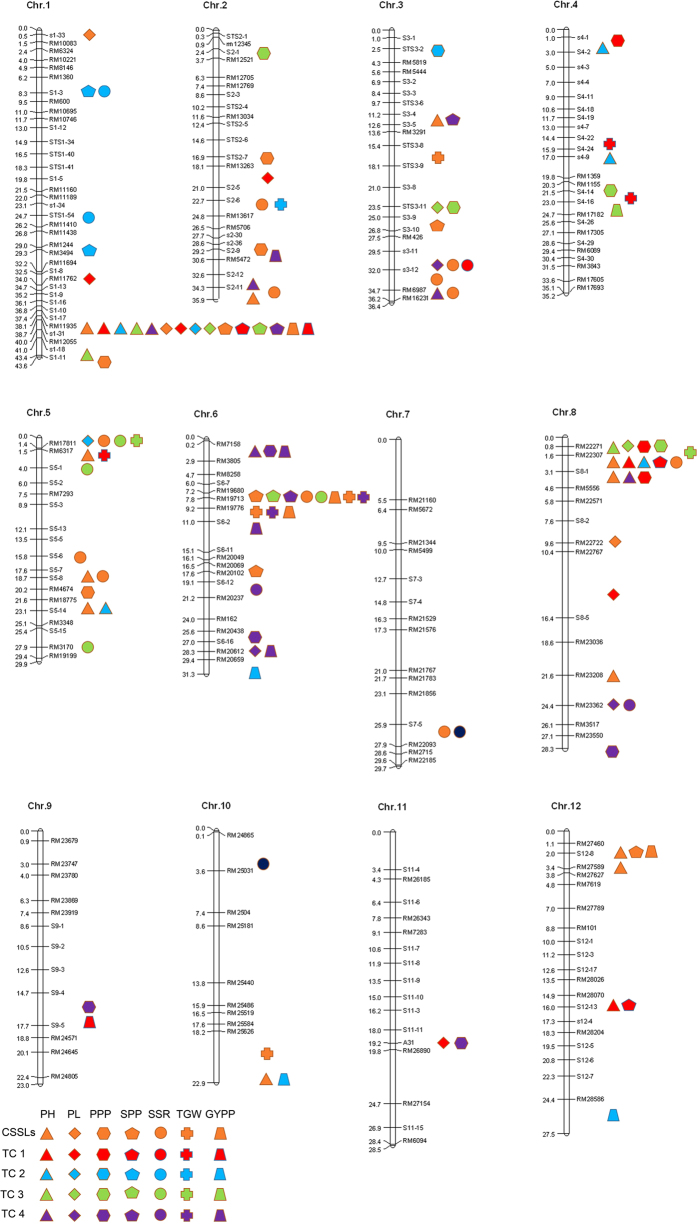


The QTLs and HLs analyses were conducted for seven yield-related traits in 2009, 2011, and 2015. Thus, it is time-consuming and laborious to detect stable and useful QTLs and HLs. Encouragingly, we identified some stable QTLs in all 3 years, which suggested the possibility of using a marker-based strategy to identify superior parents of inter-subspecies hybrids. Among them, a large-effect QTL was located in bin x36, the region harboring the rice ‘green revolution’ gene *sd1*^30^. The *SD1* gene encodes a defective enzyme in the GA-biosynthetic pathway. The utilization of *sd1* in cereal crops has enhanced resistance to lodging and increased the harvest index, ultimately increasing yield and production. We also found some HLs in more than one TC population, at identical sites or at nearby intervals on the same chromosomes. Interestingly, some detected QTLs were also identified in TC populations. These results showed that there is indeed a significant relationship between TC hybrid and the parental lines, which is consistent with phenotypic correlation analyses. Therefore, mapping of QTLs and HLs is a feasible strategy that should receive more attention. As shown in Fig. 4, we observed that some QTLs and HLs had a clustered distribution (near RM11935 on chromosome 1, near RM17811 on chromosome 5, near RM19713 on chromosome 6, and near RM22271 on chromosome 8). These QTLs and/or HLs ‘hot spots’ should be analyzed in further studies.

Determining the mechanism of heterosis is a worldwide challenge in biology, mainly because heterosis is a complex biological phenomenon controlled by multiple genes and the heterosis of different traits is affected by combinations of partially non-redundant loci. To resolve the molecular biological basis of heterosis, we should concentrate on particular traits, such as yield-related traits, and identify their major HLs. Using near isogenic lines or CSSLs containing these QTLs as materials could remove the genetic background noise. The development of plant genomics and cloning of heterosis-related QTLs or genes will have important driving effects on studies of the mechanisms of heterosis.

## Materials and Methods

### Plant materials

The population comprising 128 CSSLs was developed from a cross between the *japonica* cultivar Nipponbare as the donor, and the *indica* cultivar 9311 as the recurrent parent. Each CSSL line was genotyped, and a high-quality physical map of ultrahigh-density single nucleotide polymorphisms (SNPs) based on whole-genome re-sequencing data was constructed^31^. Each CSSL was genotyped on the basis of the physical locations and genotypes of SNPs. A physical map of the 128 CSSLs was constructed and was used for QTL mapping of yield-related traits in rice. Three photo-thermo-sensitive-genic male sterile (PTGMS) lines (Guangzhan63-4s, 036s, and Lian99s) were selected as females and were crossed with the 128 CSSLs to generate the following four TCs: TC 1 (Guangzhan63-4s × CSSLs in 2008), TC 2 (Guangzhan63-4s × CSSLs in 2010), TC 3 (036s × CSSLs in 2014), and TC 4 (Lian99s × CSSLs in 2014). The F_1_ hybrids of Guangzhan63-4s × 9311, 036s × 9311, and Lian99s × 9311, along with the two parents, were included in the yield evaluations.

### Phenotypic evaluation

The TC populations were generated in the summers of 2008, 2010, and 2014, and the phenotypic evaluations were conducted in the summers of 2009, 2011, and 2015 at the experimental field of Yangzhou University (Jiangsu, China). Four TCs, the CSSLs, the F_1_ hybrids (Guangzhan63-4s × 9311, 036s × 9311, and Lian99s × 9311) and the parental lines (9311 and Nipponbare) were planted with two replications. Each plot consisted of four rows, and each row had 10 plants. Materials were sown on May 14^th^ in each year. Thirty-day-old seedlings were transplanted to an experimental field in which the plants were spaced at a distance of (26.4 + 16.5) × 13.2 cm. The recommended agronomic practices for hybrid rice were applied in the experimental plots: the middle five plants in the central row of each plot were used for data collection. The seven quantitative traits investigated were PH, PL, PPP, SPP, SSR, GYPP, and TGW.

### Data analysis, QTLs and HLs mapping

The means and standard deviations of the values of the seven phenotypic traits among TCs and CSSLs were analyzed using Microsoft Excel 2013. Statistical analyses, including correlation analyses, *t*-tests, and K–S tests were conducted using SPSS version 13.0 (SPSS, Inc., Chicago, IL, USA). Box-plots of the seven traits among TCs and CSSLs were drawn using SigmaPlot version 10.0 (Systat Software, Inc., CA, USA).

Analyses of QTLs and HLs were conducted as described previously^31^. The following multiple linear model was used:


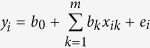


where *y*_*i*_ is the mean value of the *i*th line of the CSSL population and the heterosis value (HV) of the *i*th line of the TC population. The definition of HV is HV = (F_1_ − CK)/CK, where F_1_ is the mean value of the *i*th line of the TC population and CK is the mean value of “PTGMS × 9311”. *b*_*0*_ is the overall mean of the population, *m* is the total number of bins in the whole genome, and *b*_*k*_ is the main effect associated with bin *k*. *x*_*ik*_ is an indicator variable, denoting *x*_*ik*_ = 1 for the donor parent bin and *x*_*ik*_ = −1 for the recurrent parent bin. *e*_*i*_ denotes the residual error following a normal distribution. Contributions of the target bins to phenotypic variation were estimated using a multiple linear regression analysis with the stepwise option in the REG procedure of the SAS software package. Analysis of variance (ANOVA) was conducted for hybrids, where variance components, broad sense heritability, and narrow sense heritability were estimated according to the model described by Mo^32^ (Table S14).

## Additional Information

**How to cite this article**: Tao, Y. *et al*. Exploitation of heterosis loci for yield and yield components in rice using chromosome segment substitution lines. *Sci. Rep.* **6**, 36802; doi: 10.1038/srep36802 (2016).

**Publisher’s note:** Springer Nature remains neutral with regard to jurisdictional claims in published maps and institutional affiliations.

## Supplementary Material

Supplementary Information

## Figures and Tables

**Table 1:** Correlation coefficients between seven traits in CSSLs and TC 1 in 2009.

Traits	Population	PH	PL	PPP	SPP	SSR	GYPP
PL	CSSLs	0.564**					
	TC 1	0.401**					
PPP	CSSLs	−0.232	−0.173				
	TC 1	−0.245	−0.101				
SPP	CSSLs	0.469**	0.396**	−0.135			
	TC 1	0.514**	0.737**	−0.152			
SSR	CSSLs	0.518**	0.201	−0.110	0.143		
	TC 1	0.202	0.398**	−0.240	0.391**		
GYPP	CSSLs	0.596**	0.480**	0.184	0.553**	0.495**	
	TC 1	0.521**	0.631**	−0.012	0.665**	0.581**	
TGW	CSSLs	0.204	−0.201	0.047	−0.161	0.033	−0.071
	TC 1	0.077	0.105	−0.146	0.102	0.270	0.185

^*^Correlation significant at the 0.05 level (two-tailed).

^**^Correlation significant at the 0.01 level (two-tailed).

**Table 2:** Correlation coefficients between seven traits in CSSLs and TC 2 in 2011.

Traits	Population	PH	PL	PPP	SPP	SSR	GYPP
PL	CSSLs	0.187*					
	TC 2	0.523**					
PPP	CSSLs	−0.172	−0.275**				
	TC 2	0.039	0.163				
SPP	CSSLs	−0.041	0.125	−0.067			
	TC 2	0.372**	0.284**	0.348**			
SSR	CSSLs	0.005	0.100	−0.111	−0.353**		
	TC 2	0.324**	0.374**	−0.168	0.263**		
GYPP	CSSLs	0.191*	−0.006	0.220*	0.047	0.044	
	TC 2	0.218*	0.240*	0.459**	−0.013	0.257**	
TGW	CSSLs	−0.037	−0.080	0.116	−0.084	−0.031	0.362**
	TC 2	0.014	−0.071	0.196*	0.015	0.052	0.301**

^*^Correlation significant at the 0.05 level (two-tailed).

^**^Correlation significant at the 0.01 level (two-tailed).

**Table 3:** Correlation coefficients between seven traits in CSSLs, TC 3 and TC 4 in 2015.

Traits	Population	PH	PL	PPP	SPP	SSR	GYPP
PL	CSSLs	0.507**					
	TC 3	0.373**					
	TC 4	0.189*					
PPP	CSSLs	−0.278**	0.051				
	TC 3	−0.066	0.208*				
	TC 4	−0.061	0.326**				
SPP	CSSLs	0.448**	−0.153	−0.214*			
	TC 3	−0.129	0.022	0.302**			
	TC 4	0.405**	0.059	0.059			
SSR	CSSLs	0.155	0.045	−0.173*	0.002		
	TC 3	0.369**	0.179	0.015	−0.151		
	TC 4	0.083	−0.022	−0.075	0.064		
GYPP	CSSLs	0.179*	−0.058	0.259**	0.234**	0.210*	
	TC 3	−0.060	−0.088	−0.002	−0.066	−0.010	
	TC 4	0.268**	0.159	0.222**	0.322**	0.122	
TGW	CSSLs	0.100	0.058	−0.110	−0.067	0.048	0.248**
	TC 3	0.037	0.003	−0.314**	−0.303**	0.020	−0.130
	TC 4	0.132	0.347**	−0.107	−0.149	0.133	0.310

^*^Correlation significant at the 0.05 level (two-tailed).

^**^Correlation significant at the 0.01 level (two-tailed).

**Table 4:** Correlation coefficients between seven traits in each CSSL and its corresponding TC.

	**PH**	**PL**	**PPP**	**SPP**	**SSR**	**GYPP**	**TGW**
CSSLs – TC 1	0.761**	0.464**	−0.113	0.421**	0.077	0.448**	0.513**
CSSLs – TC 2	0.836**	0.092	−0.119	0.832**	0.049	0.288**	0.397**
CSSLs – TC 3	0.666**	0.062	0.022	0.048	0.051	0.252**	0.470**
CSSLs – TC 4	0.561**	0.146	0.023	0.264**	0.022	−0.020	0.327**

^*^Correlation significant at the 0.05 level (two-tailed).

^**^Correlation significant at the 0.01 level (two-tailed).

**Table 5:** Genetic parameters estimates for seven traits.

**Trait**	***h***_***b***_^***2***^**(%)**	***h***_***n***_^***2***^**(%)**	**Additive effect**	**Dominant effect**	**Degree of dominance**
PH	79.85	63.29	63.29	16.56	0.51
PL	51.22	33.95	33.95	17.27	0.71
SPP	43.49	9.72	9.72	33.77	1.86
PPP	21.47	5.21	5.21	16.26	1.77
SSR	44.91	29.38	29.38	15.53	0.73
GYPP	75.50	60.66	60.66	14.84	0.51
TGW	76.75	61.42	61.42	15.33	0.53
