# Supplementary material for: Exploitation of heterosis loci for yield and yield components in rice using chromosome segment substitution lines
Source: Sci Rep. 2016 Nov 11;6:36802. doi: 10.1038/srep36802 (PMC5105071; doi:10.1038/srep36802)
Supplement: Supplementary Information [file srep36802-s1.pdf]

Exploitation of heterosis loci for yield and yield components in rice using chromosome segment substitution lines

Yajun Tao[+,1], Jinyan Zhu[+,2], Jianjun Xu[1], Liujun Wang[1], Houwen Gu[1], Ronghua Zhou[1], Zefeng Yang[1], Yong Zhou[*,1], Guohua Liang[*,1]

Table S1. K-S test for seven traits in CSSLs and TC 1 in 2009

Asymp. Sig. (2-tailed)	PH	PL	SPP	SSR	PPP	GYPP	TGW
CSSLs	0.000	0.209	0.944	0.000	0.691	0.484	0.664
TC 1	0.000	0.924	0.211	0.066	0.013	0.969	0.527

P≥0.05, sample is normal distribution.

Table S2. K-S test for seven traits in CSSLs and TC 2 in 2011

Asymp. Sig. (2-tailed)	PH	PL	SPP	SSR	PPP	GYPP	TGW
CSSLs	0.000	0.161	0.322	0.044	0.429	0.294	0.000
TC 2	0.000	0.000	0.586	0.003	0.141	0.463	0.691

P≥0.05, sample is normal distribution.

Table S3. K-S test for seven traits in CSSLs and TC 3 and TC 4 in 2015

Asymp. Sig. (2-tailed)	PH	PL	SPP	SSR	PPP	GYPP	TGW
CSSLs	0.000	0.000	0.363	0.001	0.017	0.390	0.778
TC 3	0.001	0.656	0.670	0.001	0.006	0.463	0.535
TC 4	0.001	0.229	0.750	0.001	0.008	0.808	0.479

P≥0.05, sample is normal distribution.

Table S4. Phenotypic variation of seven yield-related traits in CSSLs and F_1 hybrids in 2009

Traits	9311	CSSLs		Guanzhan63-4s×9311	TC 1	
	Mean ±SD	Range	Mean ±SD	Mean ±SD	Range	Mean ±SD
PH	139.66 ±2.05	103.57-189.35	139.03 ±13.62	145.38 ±3.46	104.75-174.38	142.88 ±8.50**
PL	25.88 ±0.96	23.78-29.38	26.02 ±1.02	27.38 ±1.09	24.70-32.71	28.74 ±1.24**
PPP	5.70 ±1.12	4.17-6.97	5.58 ±0.47	7.13 ±0.99	5.38-14.5	7.24 ±0.91**
SPP	232.53 ±12.48	174.88-318.73	235.39 ±23.91	254.88 ±10.71	149.88-368.63	257.44 ±26.97**
SSR	0.93 ±0.02	0.52-0.97	0.89 ±0.05	0.79 ±0.03	0.64-0.98	0.83 ±0.08**
GYPP	29.37 ±1.97	16.26-43.60	28.75 ±4.09	31.28 ±2.38	17.98-48.56	32.33 ±5.11**
TGW	28.66 ±0.27	25.18-31.44	28.45 ±1.11	28.30 ±0.55	25.66-31.79	28.40 ±0.85

* p≤0.05, ** p≤0.01. The T-test were conducted between means of CSSLs and TC 1.

Table S5. Phenotypic variation of seven yield-related traits in CSSLs and F_1 hybrids in 2011

Traits	9311	CSSLs		Guanzhan63-4s×9311	TC 2	
	Mean ±SD	Range	Mean ±SD	Mean ±SD	Range	Mean ±SD
PH	115.05±1.66	84.20-184.20	122.85±15.74	120.45±9.25	105.50-150.90	120.45±9.25
PL	24.02±0.89	21.66-28.52	24.19±1.74	24.33±1.27	24.33-47.65	26.87±2.44**
PPP	6.00±1.02	4.20-9.80	6.87±1.13	5.85±1.21	4.71-14.50	7.35±1.44*
SPP	266.60±25.31	195.20-379.00	266.58±32.82	254.63±27.59	184.00-384.00	259.94±32.54
SSR	0.93±00.47	0.65-0.98	0.90±0.50	0.89±0.03	0.58-0.97	0.89±0.81
GYPP	16.8±1.29	5.40-38.60	24.15±6.00	19.90±1.38	10.40-52.10	26.43±7.40*
TGW	30.11±2.11	24.60-34.70	28.87±3.93	30.68±1.22	25.10-30.60	28.39±0.94

* p≤0.05, ** p≤0.01. * p≤0.05, ** p≤0.01. The T-test were conducted between means of CSSLs and TC 2.

Table S6. Phenotypic variation of seven yield-related traits in CSSLs and F_1 hybrids in 2015

Traits	9311	CSSLs		036s×9311	TC 3		Lian99s×9311	TC 4	
	Mean ±SD	Range	Mean ±SD	Mean ±SD	Range	Mean ±SD	Mean ±SD	Range	Mean ±SD
PH	123.29±1.04	84.86-176.71	122.97±12.03	120.61±2.33	106.29-144.00	118.67±6.28**	118.43±3.15	104.00-145.14	118.85±7.37**
PL	24.41±0.77	19.51-28.4	24.78±8.97	27.36±1.21	24.75-32.30	27.60±1.12**	28.16±0.59	21.60-29.37	27.19±1.21**
PPP	4.43±1.02	3.00-6.71	4.43±0.56	6.14±0.34	4.14-11.5	5.99±1.13**	4.71±1.14	3.57-15.00	5.82±1.46**
SPP	233.00±10.56	175.33-324.4	233.93±30.58	254.63±29.03	105.60-301.5	218.31±37.07**	244.00±27.42	132.80-332.14	241.38±32.08*
SSR	0.97±0.02	0.65-0.98	0.94±0.04	0.81±0.07	0.71-0.98	0.93±0.05*	0.77±0.02	0.30-0.99	0.93±0.08*
GYPP	30.21±2.21	12.62-42.41	24.98±3.94	24.47±2.42	11.58-41.26	23.31±5.36**	24.37±3.24	12.90-52.03	27.70±5.94**
TGW	32.69±0.33	27.63-35.31	31.71±1.34	27.79±0.76	25.64-31.56	28.80±1.06**	28.38±0.92	24.07-30.84	27.99±1.09**

* p≤0.05, ** p≤0.01. * p≤0.05, ** p≤0.01. The T-test were conducted among means of CSSLs, TC 3 and TC 4.

Table S7. QTLs and HLs mapped for plant height in CSSLs and TCs

Years	QTLs/HLs	Bins	Chr.	Interval/bp	Size of the Interval/bp	Partial R-Square	F
2009 CSSLs	*qPH1-1*	x36	1	39868630-40660285	791655	0.6295	214.05
	qPH2-1	x107	2	36017977-36777825	759848	0.0247	19.46
	qPH2-2	x108	2	36777825-36823111	45286	0.0176	18.39
	qPH5-1	x203	5	18561151-18983458	422307	0.0223	20.33
	qPH8-1	x278	8	2797908-3336084	538176	0.1133	55.08
	qPH8-2	x302	8	21869706-22021453	151747	0.0108	12.4
	qPH12-1	x389	12	1285320-2287169	1001849	0.044	30.12
	qPH12-2	x390	12	2287169-3113546	826377	0.0337	18.71
2011 CSSLs	*qPH1-1*	x36	1	39868630-40660285	791655	0.7134	298.65
	qPH3-1	x129	3	12745775-12844058	98283	0.0063	7.94
	qPH5-2	x192	5	2049179-2208395	159216	0.0095	9.18
	qPH8-1	x278	8	2797908-3336084	538176	0.0385	22.54
	qPH10-1	x362	10	22335288-22517954	182666	0.0058	7.69
	qPH12-1	x389	12	1285320-2287169	1001849	0.0386	27.68
	qPH12-2	x390	12	2287169-3113546	826377	0.0466	23.12
2015 CSSLs	*qPH1-1*	x36	1	39868630-40660285	791655	0.6717	251.69
	qPH2-1	x107	2	36017977-36777825	759848	0.0120	10.51
	qPH2-2	x108	2	36777825-36823111	45286	0.0133	12.86
	qPH5-3	x216	5	23739776-23953127	213351	0.0374	25.07
	qPH8-3	x279	8	3336084-4225627	889543	0.1104	61.81
2009 TC 1	*hPH1-1*	x31	1	37528985-37872430	343445	0.3302	62.11
	hPH1-2	x33	1	38016171-39172107	1155936	0.1178	47.63
	hPH1-3	x35	1	39372177-39868630	496453	0.0445	23.40
	hPH1-4	x36	1	39868630-40660285	791655	0.0303	13.48
	hPH1-5	x50	1	44909213-45040476	131263	0.0378	26.06
	hPH1-6	x51	1	45040476-45064769	24293	0.0482	45.56
	hPH8-1	x278	8	2797908-3336084	538176	0.2455	72.31
	hPH12-1	x395	12	16467362-19737821	3270459	0.0201	11.51
2011 TC 2	*hPH1-4*	x36	1	39868630-40660285	791655	0.8140	446.33
	hPH4-1	x158	4	2621462-4805697	2184235	0.0148	12.83
	hPH4-2	x165	4	17449792-18051474	601682	0.0091	8.50
	hPH5-1	x216	5	23739776-23953127	213351	0.0365	24.62
	hPH8-1	x278	8	2797908-3336084	538176	0.0205	15.87
2015 TC 3	*hPH1-4*	x36	1	39868630-40660285	791655	0.1106	109.00
	hPH1-6	x49	1	44583788-44909213	325425	0.0149	18.45
	hPH8-2	x276	8	2449919-2492172	42253	0.7717	395.46
2015 TC 4	*hPH1-4*	x36	1	39868630-40660285	791655	0.0943	117.40
	hPH2-1	x103	2	34840017-35020986	180969	0.0034	10.66
	hPH3-1	x153	3	36134582-37025404	890822	0.0225	36.42
	hPH6-1	x228	6	1171547-1238283	66736	0.4020	81.33
	hPH8-3	x279	8	3336084-4225627	889543	0.0069	14.93

Table S8. QTLs and HLs mapped for panicle length in CSSLs and TCs

Years	QTLs/HLs	Bins	Chr.	Interval/bp	Size of the Interval/bp	Partial R-Square	F
2009 CSSLs	*qPL1-1*	x36	1	39868630-40660285	791655	0.3628	71.75
	qPL8-1	x290	8	9529984-9684529	154545	0.0546	11.71
2011 CSSLs	*qPL8-2*	x304	8	24174829-24278714	103885	0.0627	8.02
2015 CSSLs	*qPL1-2*	x1	1	1-2366727	2366726	0.9768	5186.60
	qPL1-1	x36	1	39868630-40660285	791655	0.0029	17.21
2009 TC 1	*hPL1-1*	x22	1	34025701-34206952	181251	0.0920	18.44
	hPL1-2	x36	1	39868630-40660285	791655	0.0892	15.67
	hPL2-1	x74	2	19710693-19742916	32223	0.1044	14.69
	hPL8-1	x294	8	14833918-14970068	136150	0.0679	15.17
	hPL11-1	x380	11	19120157-19494142	373985	0.1003	15.76
2011 TC 2	*hPL1-2*	x36	1	39868630-40660285	791655	0.0337	13.37
	hPL5-1	x187	5	1-526890	526889	0.2995	43.62
	hPL5-2	x188	5	526890-1258372	731482	0.4147	146.53
2015 TC 3	*hPL1-2*	x36	1	39868630-40660285	791655	0.0179	17.10
	hPL3-1	x140	3	24417950-24936333	518383	0.0237	19.89
	hPL8-2	x275	8	861272-2449919	1588647	0.8384	606.83
2015 TC 4	*hPL3-2*	x145	3	30831094-31748972	917878	0.1341	209.97
	hPL3-3	x146	3	31748972-32219641	470669	0.0206	11.49
	hPL6-1	x250	6	28410389-28439277	28888	0.0999	23.91
	hPL6-2	x251	6	28439277-28565216	125939	0.2141	89.27
	hPL8-3	x307	8	24929962-25156456	226494	0.0136	25.90

Table S9. QTLs and HLs mapped for panicles per plant in CSSLs and TCs

Years	QTLs/HLs	Bins	Chr.	Interval/bp	Size of the Interval/bp	Partial R-Square	F
2009 CSSLs	*qPPP5-1*	x214	5	21630309-23222854	1592545	0.1184	16.93
2011 CSSLs	*qPPP2-1*	x67	2	17154017-17168425	14408	0.0696	8.98
2015 CSSLs	*qPPP1-1*	x51	1	45040476-45064769	24293	0.0285	7.83
	qPPP2-2	x97	2	29526471-30607343	1080872	0.1389	539.182
2009 TC 1	*hPPP4-1*	x157	4	1897737-2621462	723725	0.0461	11.47
	hPPP8-1	x275	8	861272-2449919	1588647	0.0564	12.94
	hPPP8-2	x279	8	3336084-4225627	889543	0.3989	83.61
2011 TC 2	*hPPP3-1*	x117	3	3763846-4248835	484989	0.1928	24.37
	hPPP4-2	x173	4	21489179-21505317	16138	0.0727	10.00
2015 TC 3	*hPPP2-2*	x55	2	3061234-3440496	379262	0.0984	20.83
	hPPP3-2	x140	3	24417950-24936333	518383	0.1650	29.84
	hPPP8-1	x275	8	861272-2449919	1588647	0.1934	28.06
2015 TC 4	*hPPP6-1*	x228	6	1171547-1238283	66736	0.1000	19.03
	hPPP6-2	x248	6	27601476-28029327	427851	0.0745	15.94
	hPPP8-3	x314	8	28393571-28530027	136456	0.2693	44.60
	hPPP9-1	x322	9	15454333-15468255	13922	0.0782	19.17
	hPPP11-1	x380	11	19120157-19494142	373985	0.0588	16.36

Table S10. QTLs and HLs mapped for spikelets per panicle in CSSLs and TCs

Years	QTLs/HLs	Bins	Chr.	Interval/bp	Size of the Interval/bp	Partial R-Square	F
2009 CSSLs	*qSPP1-1*	x36	1	39868630-40660285	791655	0.1886	29.28
	qSPP6-1	x240	6	17605329-20704751	3099422	0.1077	19.13
2011 CSSLs	*qSPP1-1*	x36	1	39868630-40660285	791655	0.0849	11.13
	qSPP3-1	x142	3	25069454-28348560	3279106	0.0669	9.39
2015 CSSLs	*qSPP1-1*	x36	1	39868630-40660285	791655	0.1759	26.26
	qSPP6-2	x233	6	7814673-9668398	1853725	0.0597	9.53
	qSPP12-1	x389	12	1285320-2287169	1001849	0.0487	8.23
2009 TC 1	*hSPP1-1*	x36	1	39868630-40660285	791655	0.1381	20.19
	hSPP8-1	x278	8	2797908-3336084	538176	0.1172	19.68
	hSPP12-1	x395	12	16467362-19737821	3270459	0.0732	13.51
2011 TC 2	*hSPP1-2*	x5	1	8349996-8805066	455070	0.1078	14.40
	hSPP1-3	x17	1	29314891-30700072	1385181	0.0467	7.09
2015 TC 3	*hSPP1-1*	x36	1	39868630-40660285	791655	0.0218	10.54
	hSPP6-1	x232	6	6942384-7814673	872289	0.0657	18.99
	hSPP6-2	x233	6	7814673-9668398	1853725	0.0612	15.26
2015 TC 4	*hSPP1-1*	x36	1	39868630-40660285	791655	0.0477	9.77
	hSPP3-1	x128	3	12672040-12745775	73735	0.1277	17.71
	hSPP6-1	x232	6	6942384-7814673	872289	0.0628	16.64
	hSPP6-2	x233	6	7814673-9668398	1853725	0.0364	8.48

Table S11. QTLs and HLs mapped for seed setting rate in CSSLs and TCs

Years	QTLs/HLs	Bins	Chr.	Interval/bp	Size of the Interval/bp	Partial R-Square	F
2009 CSSLs	*qSSR3-1*	x146	3	31748972-32219641	470669	0.0460	11.72
	qSSR3-2	x147	3	32219641-34963314	2743673	0.1597	60.44
	qSSR3-3	x151	3	35786915-36109587	322672	0.2399	39.77
	qSSR5-1	x200	5	16633075-18486326	1853251	0.0325	13.57
	qSSR5-2	x203	5	18561151-18983458	422307	0.0675	14.16
	qSSR6-1	x232	6	6942384-7814673	872289	0.0659	15.44
	qSSR8-1	x277	8	2492172-2797908	305736	0.1013	19.22
2011 CSSLs	*qSSR2-1*	x105	2	35094728-35514634	419906	0.1459	20.49
	qSSR3-4	x154	3	37025404-37068592	43188	0.1016	18.86
	qSSR3-5	x155	3	37068592-37257345	188753	0.1171	18.90
	qSSR5-3	x188	5	526890-1258372	731482	0.0284	7.73
2015 CSSLs	*qSSR2-2*	x85	2	23918114-24635764	717650	0.0575	20.08
	qSSR3-4	x154	3	37025404-37068592	43188	0.1825	38.16
	qSSR3-5	x155	3	37068592-37257345	188753	0.2338	37.54
	qSSR6-1	x232	6	6942384-7814673	872289	0.0261	9.79
	qSSR7-1	x272	7	26590657-27431495	840838	0.0192	7.62
	qSSR8-2	x278	8	2797908-3336084	538176	0.0690	20.80
2009 TC 1	*hSSR3-1*	x146	3	31748972-32219641	470669	0.1525	22.67
2011 TC 2	*hSSR1-1*	x5	1	8349996-8805066	455070	0.1062	12.47
	hSSR1-2	x15	1	25646290-29042972	3396682	0.0201	13.01
2015 TC 3	*hSSR5-1*	x188	5	526890-1258372	731482	0.0001	49.69
	hSSR5-2	x195	5	3589806-4050901	461095	0.0001	10.70
	hSSR5-3	x196	5	4050901-5327078	1276177	0.0001	17.10
	hSSR5-4	x225	5	28689993-29833991	1143998	0.0001	80.52
	hSSR6-1	x232	6	6942384-7814673	872289	0.0001	193.31
	hSSR6-2	x233	6	7814673-9668398	1853725	0.0001	59.11
2015 TC 4	*hSSR6-3*	x241	6	20704751-21140686	435935	0.0890	17.38
	hSSR7-1	x272	7	26590657-27431495	840838	0.0454	9.50
	hSSR8-1	x307	8	24929962-25156456	226494	0.0987	16.94
	hSSR10-1	x334	10	3590051-4462767	872716	0.0590	17.05

Table S12. QTLs and HLs mapped for thousand grain weight in CSSLs and TCs

Years	QTLs/HLs	Bins	Chr.	Interval/bp	Size of the Interval/bp	Partial R-Square	F
2009 CSSLs	*qTGW3-1*	x135	3	16634855-22427809	5792954	0.0853	11.75
2011 CSSLs	*qTGW6-1*	x232	6	6942384-7814673	872289	0.1438	20.15
2015 CSSLs	*qTGW6-1*	x232	6	6942384-7814673	872289	0.0470	14.78
	qTGW6-2	x234	6	9668398-9927733	259335	0.0378	13.14
	qTGW10-1	x357	10	21115895-21582159	466264	0.1451	20.87
2009 TC 1	*hTGW5-1*	x192	5	2049179-2208395	159216	0.1465	21.62
2011 TC 2	*hTGW2-1*	x85	2	23918114-24635764	717650	0.0083	8.74
	hTGW4-1	x163	4	16718989-17316368	597379	0.3061	44.99
	hTGW4-2	x178	4	23642092-25694141	2052049	0.1390	25.29
	hTGW4-3	x179	4	25694141-30907937	5213796	0.1089	24.41
2015 TC 3	*hTGW5-1*	x188	5	526890-1258372	731482	0.4667	727.83
	hTGW8-1	x276	8	2449919-2492172	42253	0.4589	99.22
2015 TC 4	*hTGW6-1*	x232	6	6942384-7814673	872289	0.0744	27.34
	hTGW6-2	x233	6	7814673-9668398	1853725	0.0671	20.11
	hTGW6-3	x234	6	9668398-9927733	259335	0.0015	7.81

Table S13. QTLs and HLs mapped for grain yield per plant in CSSLs and TCs

Years	QTLs/HLs	Bins	Chr.	Interval/bp	Size of the Interval/bp	Partial R-Square	F
2009	*qGYPP1-1*	x36	1	39868630-40660285	791655	0.2068	32.85
CSSLs	*qGYPP8-1*	x278	8	2797908-3336084	538176	0.1244	23.26
2011 CSSLs	*qGYPP6-1*	x232	6	6942384-7814673	872289	0.1438	20.15
2015 CSSLs	*qGYPP6-2*	x234	6	9668398-9927733	259335	0.1597	23.38
2009 TC 1	*hGYPP1-1*	x36	1	39868630-40660285	791655	0.1314	19.07
	hGYPP8-1	x278	8	2797908-3336084	538176	0.1244	23.26
	hGYPP9-1	x323	9	15468255-15567417	99162	0.8010	21.77
2011 TC 2	*hGYPP6-1*	x257	6	31240409-32113038	872629	0.0608	8.00
	hGYPP10-1	x362	10	22335288-22517954	182666	0.0799	9.83
	hGYPP12-1	x400	12	25640209-25879909	239700	0.0995	11.27
2015	*hGYPP4-1*	x176	4	23285463-23315504	30041	0.1178	15.62
TC 3	*hGYPP8-2*	x276	8	2449919-2492172	42253	0.1183	17.97
2015 TC 4	*hGYPP2-1*	x98	2	30607343-31516121	908778	0.0527	7.88
	hGYPP6-2	x228	6	1171547-1238283	66736	0.0753	9.85
	hGYPP6-3	x250	6	28410389-28439277	28888	0.0472	7.45
	hGYPP8-3	x307	8	24929962-25156456	226494	0.0765	10.83

Table S14 Variance analysis of seven traits

Source	*df*	Anova SS	Mean Square	EMS
Hybrid	*pq-1*			
CSSLs	*q-1*	SS_m	MS_m	$\sigma_e^2+r\sigma_{fm}^2+rp\sigma_m^2$
PTGMS	*p-1*	SS_f	MS_f	$\sigma_e^2+r\sigma_{fm}^2+rq\sigma_f^2$
PTGMS × CSSLs	*(p-1)(q-1)*	SS_{fm}	MS_{fm}	$\sigma_e^2+r\sigma_{fm}^2$
Error	*pq(r-1)*	SS_e	MS_e	σ_e^2
Total	*pqr-1*	SS_T		

1. $\sigma_{d1}^2=2\sigma_f^2$
2. $\sigma_{d2}^2=2\sigma_m^2$
3. $\sigma_G^2=\sigma_f^2+\sigma_m^2+\sigma_{fm}^2$
4. $\sigma_p^2=\sigma_G^2+\sigma_e^2$
5. $h_b^2=\sigma_G^2/\sigma_p^2$
6. $h_n^2=(\sigma_f^2+\sigma_m^2)/\sigma_p^2$
7. $\sigma_h^2=\sigma_{fm}^2$
8. $Degree\ of\ dominance=\sqrt{\sigma_h^2/(\sigma_f^2+\sigma_m^2)}$

p and *q* donate the number of CSSLs and PTGMS.

r donates the repeat of every hybrid.

σ_{d1}^2 donates the variance of PTGMS.

σ_{d2}^2 donates the variance of CSSLs.

σ_G^2 donates the genotypic variance of F_1.

σ_p^2 donates the phenotypic variance of F_1.

h_b^2 donates the broad sense heritability.

h_n^2 donates the narrow sense heritability.

σ_h^2 donates the variance of PTGMS × CSSLs.
